# Screening for cellulases and preliminary optimisation of glucose tolerant β-glucosidase production and characterisation

**DOI:** 10.1080/21501203.2022.2155261

**Published:** 2022-12-17

**Authors:** Nivisti Singh, Bruce Sithole, Roshini Govinden

**Affiliations:** a Discipline of Microbiology, School of Life Sciences, Westville Campus, University of KwaZulu-Natal, Durban, South Africa; b Discipline of Engineering, Howard Campus, University of KwaZulu-Natal, Durban, South Africa; c Biorefinery Industry Development Facility, Council for Scientific and Industrial Research, Durban, South Africa

**Keywords:** Endoglucanases, exoglucanases, β- glucosidases, glucose tolerant, cellulases

## Abstract

The search for a novel microbial producer of cellulases including a glucose tolerant β-glucosidase is a challenge as most are inhibited by their product glucose. This study aims to screen for cellulolytic fungi using qualitative and quantitative screening methods. Primary screening revealed 34 of 46 fungal isolates with β-glucosidase activity. Eleven and 13 of these also displayed endoglucanase and exoglucanase activities, respectively. During secondary screening, this number was reduced to 26 β-glucosidase producers with 13 also having endoglucanase and exoglucanase activities. Isolate C1 displayed enhanced production of β-glucosidases in the presence of 0.05 M glucose (69% higher activity). Optimisation of growth conditions for β-glucosidase production by one variable at a time experiments improved production for (isolates) PS1 (64%), MB5 (84%), and C2 (69%). Isolate PS1 identified as *Chaetomella* sp. BBA70074 displayed the highest tolerance to glucose, retaining 10% of β-glucosidase activity in the presence of 0.8 M glucose. Tolerance to glucose increased to 14% when produced under optimal conditions. β-Glucosidase had a molecular weight of 170 kDa with a pH and temperature optima of 6 and 70°C, respectively. Future studies will include optimisation of the production of the glucose tolerant enzyme by *Chaetomella* sp. BBA70074.

## 1. Introduction

The depletion of fossil fuels, the negative environmental impact resulting from their combustion, and the increasing energy demand have paved the way for sustainable biofuels and bioproducts from renewable resources by biorefineries (Sørensen et al. 2013). The transportation, chemical, and plastic production sectors are dependent on oil as their primary energy source. Soon biorefineries are expected to supplement, if not completely replace oil refineries by maximising the use of the tonnes of renewable biomass currently being disposed off in landfills, to produce biofuels and basic molecules as building blocks for the synthesis of chemicals and polymeric materials (Cherubini 2010; Kanakaraju et al. 2020). Not only are landfills reaching their maximum capacity, but they pose environmental and financial concerns, therefore, biomass valorisation will solve both issues of concern (Srivastava et al. 2019).

During the pulp and paper manufacturing process, paper mills generate large amounts of solid waste products referred to as pulp and paper mill sludge (PPMS). In South Africa, four to five hundred thousand wet tonnes of PPMS are produced, which makes the pulp and paper mill industry one of the highest polluting industries (Darwesh et al. 2020). Pulp and paper mill sludge contain organic and inorganic materials; however, the majority is organic material that includes lignocellulosic residuals originating from wood, the raw material used in the pulp and paper making process (Naicker et al. 2020).

Biorefineries rely upon this lignocellulosic biomass as an abundant and renewable energy source (Sørensen et al. 2013). Most biorefineries focus on hydrolysing the biomass into simple sugars that serve as feedstocks for chemical and microbial conversion to biofuels (ethanol, butanol, and hydrocarbons), different organic acids, and other higher value products (Naicker et al. 2020). Enzymatic hydrolysis is the preferred method of hydrolysis compared to chemical methods that eventually release harmful chemicals, thus increasing environmental pollution (Srivastava et al. 2019).

Enzymatic hydrolysis of lignocellulosic biomass is carried out by cellulases, a group of three different enzymes, namely, endoglucanases (1, 4-β-_D_-glucanohydrolase) which hydrolyse the β 1–4 glycosidic bonds within the cellulose chain exposing reducing and non-reducing ends, which are then acted upon by exoglucanases (1, 4-β-_D_-glucan cellobiohydrolase) releasing cellobiose and cello-oligosaccharides. In the final step β-glucosidases (β-_D_-glucopyranoside glucohydrolases) convert these products into free glucose units (Singh et al. 2016). The majority of cellulase cocktails from native fungal producers are unable to completely hydrolyse cellulose due to the low titres of β-glucosidases (Kumar and Wyman 2009; Rani et al. 2014). Darwesh et al. (2020) reported that cellulases from *Aspergillus Niger* MK543209 in a 2:1:1 (endoglucanases: exoglucanases: β-glucosidases) ratio resulted in the complete hydrolysis of cellulose to glucose.

The activity of β-glucosidase is the rate-limiting step that determines the overall rate of conversion of cellulose into glucose molecules (Srivastava et al. 2019). The main obstacles to commercialising an efficient microbial β-glucosidase include product inhibition and thermal inactivation, which reduce process/production yields, as well as the high cost of enzyme production (Yao et al. 2016). During lignocellulose biomass conversion, cellobiose is an inhibitor of cellobiohydrolases and endoglucanases; therefore, β-glucosidases are incorporated to mitigate this by hydrolysing the cellobiose to glucose; however, β-glucosidases in turn are often inhibited by their own product, which is glucose, thus making them the rate-limiting step during the hydrolysis of cellulosic biomass (Ahmed et al. 2017). It has been postulated that a glucose tolerant β-glucosidases would ensure high hydrolysis rates as they will perform efficiently even when glucose levels are high (Yao et al. 2016). Hydrolysis may also be inhibited by transglycosylation events due to the reaction being a reversible process; this occurs when the active site of the β-glucosidase is used to catalyse the formation of a glycosidic bond rather than its hydrolysis (Sørensen et al. 2013). β-Glucosidases are classified into three classes based on substrate specificity, (i) aryl-β-D-glucosidases, which have a strong affinity for aryl-β-D-glucosides; (ii) cellobiases that hydrolyse only disaccharides; and (iii) broad specificity glucosidases that exhibit activity on many substrate types and are most common (Singh et al. 2016).

Glucose tolerant β-glucosidase enzymes can be divided into three groups: (i) those that are strongly inhibited by low glucose concentrations, which are most β-glucosidases; (ii) β-glucosidases that are tolerant to low glucose concentrations but strongly inhibited by high glucose concentrations, such as glucosidases from *Aspergillus oryzae* and *Candida peltata;* and (iii) β-glucosidases that are stimulated by low but inhibited by high concentrations of glucose (Cao et al. 2015).

Microbial cellulases have become key players as biocatalysts due to their complex nature and widespread industrial applications. Both fungi and bacteria produce cellulases, however, fungi have the potential to secrete large amounts of enzymes, utilise a variety of substrates through the extracellular secretion of hydrolytic enzymes, display simpler structures, and have the ability to penetrate complex substrates (Kuhad et al. 2011). *Trichoderma reesei,* a commercial producer of cellulases, utilises sophorose as an inducer of cellulases that increases production by 2500 times as compared to cellobiose; however, production costs would be high as sopharose is expensive (Zhang et al. 2022). Challenges lie in searching for a cheaper alternative with similar effects that will reduce production costs. A recent study by de Souza et al. (2021) reported efficient hydrolysis of cellulosic material by a cellulase enzyme cocktail produced by *T. reesei* utilising wheat bran as a carbon source. These researchers also discovered that the pH during the fermentation process played a key role in reducing feedback inhibition of the enzyme, thus achieving efficient cellulose hydrolysis.

Several studies have reported on species of *Aspergillus* that express glucose tolerant β-glucosidases that are insensitive to product inhibition (Sørensen et al. 2013; Zhang et al. 2017; Srivastava et al. 2019). One of the largest sources of commercial β-glucosidases is *Aspergillus niger* (*A. niger*) sold under the name Novazym 188 (Ahmed et al. 2017).

Crude cellulase cocktails are preferred for the hydrolysis of lignocellulosic biomass in industry as they may contain the full lignocellulolytic set of enzymes avoiding the use of pure analytical-grade enzymes from different sources that are expensive and may not work as well in a cocktail compared to a native producer (Zang et al. 2018, Monteiro et al. 2020, Naicker et al. 2020; de Souza et al. 2021). Current research lies in searching for a thermophilic glucose tolerant β-glucosidase producer that prefers a slightly acidic to basic pH as well as a native cellulase producer that produces thermophilic enzymes in a cocktail with optimal and proportional activities required for the complete hydrolysis of lignocellulose to free glucose units.

Therefore, the aim of this study was to search for a cellulolytic fungal isolate with the potential to produce a crude cocktail of endoglucanases, exoglucanases, and glucose tolerant β-glucosidases by isolating fungi from the environment using the serial dilution method, screening for endoglucanase, exoglucanase, and β-glucosidase activity using quantitative and qualitative methods, and further screening of the highest β-glucosidase producers for glucose tolerance.

## 2. Materials and methods

### 2.1 Sample collection and isolation of fungal isolates

Tree bark and soil samples were collected from three locations. Tree bark samples were collected from three different trees; tree one: *Acer negrundo* (Site A: 29°49ʹ01” S 30°56ʹ41” E), tree two: *Juglans regia* (Site B: 29°49ʹ03” S 30°56ʹ29” E), and tree three: *Citrus limone* (Site C: 29°16ʹ13” S 31°22ʹ06” E). Microorganisms living in this environment are expected to produce cellulases capable of cellulose degradation (Steffen et al. 2017). Tenfold serial dilutions were performed; 0.1 ml of each dilution was plated onto potato dextrose agar (PDA) plates and incubated at 30°C for five days until fungal growth was observed. Two types of long-term stocks were prepared for all isolated fungi; 25% glycerol stocks were prepared and stored at −20°C, and mineral oil slants using PDA were stored at 4°C (Adesina and Onilude 2013).

### 2.2 Primary and secondary screening of fungi for cellulase and β-glucosidase activities

The fungal isolates were grown on PDA supplemented with 1.0 g of aesculin and 0.3 g of ferric citrate per litre and incubated at 30°C for seven days to determine β-glucosidase activity. Positive β-glucosidase activity was indicated by a black precipitate around the fungal mycelium (Kao et al. 2019). The potential of their activity was determined by measuring the diameter of the black precipitate (Pérez et al. 2011). Endoglucanase and exoglucanase (cellulases) activities were determined on a minimal agar medium (0.5 g NaCl, 1.0 g KH_2_PO_4_, 0.5 g MgSO_4._7H_2_O, 0.01 g MnSO_4._H_2_O, 0.3 g NH_4_NO_3_, 0.01 g FeSO_4._7H_2_O 12 g bacteriological agar) supplemented with 1% carboxymethyl cellulose (CMC) or avicel, respectively. These plates were inoculated with a standardised inoculum and incubated at 30°C for seven days. Thereafter, the plates were flooded with 0.1% Congo red solution for 15 minutes, followed by destaining with 1 M sodium chloride (NaCl) for 10 minutes, and were examined for zones of clearing around the point of inoculation. The enzyme activity was determined by measuring the cellulolytic index (CI) by measuring the diameter millimetre (mm) of clearing divided by the diameter (mm) of colony growth (Saini et al. 2017).

Extracellular enzyme extracts were produced using a submerged fermentation process. Crude extracellular β-glucosidases and glucanases were produced in a minimal media using the methods of Kao et al. (2019) and Adesina and Onilude (2013), respectively. Each 250 ml flask contained 25 ml of medium and three 5 mm mycelial plugs of actively growing hyphae and was incubated at 30°C for seven days at 125 rpm (New Brunswick Scientific, Incubator Shaker series, Innova 44, Germany). After incubation, the extracellular supernatant was obtained by centrifugation at 16837× g (Eppendorf Centrifuge 5418, Germany) for 10 minutes and used as the crude enzyme for quantification of enzyme activity (Kao et al. 2019).

### 2.3 Enzyme assays

The crude enzyme extracts were assayed to quantify β-glucosidase, endoglucanase, and exoglucanase activities. β-Glucosidase activity was quantified using the method of Kao et al. (2019). The reaction mixture contained 100 µl of enzyme added to 100 µl of 4 (millimolar) mM *4*-nitrophenyl-β-_D_-glucopyranoside (*4*-NPG) in 0.05 M sodium acetate buffer (pH 5.0) and incubated at 55°C for 5 min of shaking. After 5 min 600 µl of 1 M Na_2_CO_3_ was added to terminate the reaction. The absorbance was read at 410 nm using a spectrophotometer (Shimadzu UV1800, Japan). One unit of activity was defined as the amount of enzyme needed to release one µmol of phenol equivalents per minute at 55°C. All the experiments and assays were duplicated and a standard curve using 4-nitrophenol (4-NP) in sodium acetate buffer (50 mM, pH 5.0) was established (Kao et al. 2019). The Beer-Lambert equation was used to calculate enzyme activity:

$$Enzyme\;activity\;\left(U.\;ml\right)=\Delta AV/\varepsilon tv$$

Where **A** = Change in absorbance, **V** = Total volume of the reaction (ml) divided by **ε **= Molar extinction co-efficient of 4-nitrophenol (µM), **t** = reaction time (minutes), and **V** = Volume of enzyme (ml).

Endoglucanase and exoglucanase activities were quantified using the dinitrosalicylic acid (DNS) method with 1% avicel or carboxymethyl cellulose (CMC) as the substrate in 0.05 M citrate buffer (pH 5.0), respectively. The reaction mixture contained 66.6 µl of the enzyme, 600 µl of 1% avicel or CMC, incubated in a water bath (Labcon 5032 U, South Africa) for 15 minutes at 55°C. The reaction was terminated by adding 1 ml of DNS to the reaction mixture, boiled at 100°C for 5 min and the absorbance was read at 540 nm using a spectrophotometer (Shimadzu UV1800, Japan). One unit of activity was defined as the amount of enzyme needed to release one µmol of glucose equivalent per minute at 55°C (Adesina and Onilude 2013). All assays were carried out in duplicate. A standard curve was established at 595 nm using a spectrophotometer (Shimadzu UV1800, Japan) and used to calculate enzyme activity (Adesina and Onilude 2013).

### 2.4 Production of β-glucosidases in the presence of glucose for the seven highest β-glucosidase producers

The ability of isolates C1, C2, C4, MS8, MB5, PB8, and PS1 to produce β-glucosidases in the presence of glucose was determined by supplementing the minimal salt medium with different concentrations of glucose (0.05 M, 0.25 M, 0.5 M, 0.75 M, and 1 M) and incubating at 30°C, 125 rpm for five days and assaying the crude extract for enzyme activity as per section 2.3.

### 2.5 Determination of glucose tolerance of the β-glucosidases produced by the seven isolates displaying the highest β-glucosidase activities

The ability of β-glucosidases produced by isolates C1, C2, C4, MS8, MB5, PB8, and PS1 to withstand the presence of glucose was determined by the method of Ariaeenejad et al. (2020). The reaction mixture that included 100 µl of the enzyme, 100 µl of *4*-NPG, and 100 µl of glucose (0.05 M, 0.25 M, 0.5 M, 0.75 M, and 1 M) was incubated at 55°C for 5 min. The reaction was terminated by the addition of 600 µl of 1 M Na_2_CO_3_ and the absorbance was read at 410 nm using a spectrophotometer (Shimadzu UV1800, Japan).

### 2.6 Identification of fungal isolates C1, C2, C4, MS8, MB8, PS1, and MB5 and phylogenetic analysis

The fungal isolates were identified based on the sequences of the ITS2 region of 18S rRNA gene. Genomic DNA was isolated according to the manufacturer’s instructions of the ZR soil Microbe DNA Miniprep™ kit (Zymo Research, USA). The polymerase-chain reaction (PCR)

mixture consisted of 1 µl 10 mM each of forward ITS5F (5’-GGAAGTAAAAGTCGTAACAAGG-3’) and reverse ITS4R (5’- CTCCTCCGCTTATTGATATGC-3’) primer, 12.5 µl of PCR master mix (Thermo Scientific, USA), DNA template 1 µl, and 9.5 µl of nuclease free water (White et al. 1990). Amplification was conducted in a thermal cycler (Biorad, USA) as follows: initial denaturation 95°C (2 min), denaturation 95°C (30 s), annealing 53°C (45 s), and extension 72°C (1 min), and the last three steps were repeated for 25 cycles (Ramnath et al. 2014). The PCR products were then subjected to electrophoresis in a 1% agarose gel and stained with ethidium bromide (0.5 µg/ml). The PCR amplicons were sequenced by the Inqaba Biotechnology DNA Sequencing Unit and the consensus sequences submitted to the National Centre for Biotechnology Information (NCBI) blast database to determine their identities.

The 18S ITS2 rRNA sequences of the seven fungal isolates were compared to other closely related strains using BLAST and the NCBI GenBank database. Alignment and phylogenetic tree construction were done using the MEGA 11 software. The phylogenetic tree was constructed using the neighbour-joining method, and the tree was evaluated using 100 bootstrap replications (Tamura et al. 2011) (Figure 5).

### 2.8 Optimisation of enzyme production by the three isolates displaying the highest tolerance to glucose C2, MB5, and PS1

Optimisation of enzyme production for isolates C2, MB5, and PS1 was conducted by evaluating the effect of a single variable at a time and thereafter, manifesting it as a standard condition before the optimisation of the next parameter. Enzyme activity was assayed after each step to determine the optimal yield. The experiments were conducted in two independent experiments with duplicate assays from each experiment (Kao et al. 2019). The variables tested included incubation time, pH, temperature, agitation, carbon, and nitrogen sources.

To determine the optimum incubation time for enzyme production, shake flasks consisting of 25 ml of minimal salt medium were inoculated and incubated as per section 2.3. Samples were obtained every 48 hours for 12 days, and enzyme activity was assayed.

Optimum pH was determined by inoculation of a minimal salt medium with different pH buffers 0.05 M sodium acetate buffer (pH 3.0 to 5.0), 0.05 M sodium phosphate buffer (pH 6.0 to 8.0), and 0.05 M glycine-NaOH buffer (pH 9.0–10.0) incubated for their optimal time for production and thereafter assayed for enzyme activity.

The optimum incubation temperature was determined by setting up shake flask cultures with media at the optimal pH and incubating them at different temperatures (25°C, 30°C, 40°C, 50°C, 60°C, 70°C, and 80°C) at 125 rpm for the optimal time duration. Thereafter, the extracts were assayed for enzyme activity.

To determine the optimum agitation rate for fermentation, broth media with optimal pH were inoculated and incubated at the optimal temperature at different agitation rates (100, 125, 150, 175, and 200 rpm), and thereafter assayed for enzyme activity.

To maximise enzyme production, various carbon and nitrogen sources were supplemented into the standard minimal medium at optimal pH in section 2.3. The following carbon (1%) lactose, maltose, glucose, sucrose, and glycerol and nitrogen sources (1%) casein, glycine, yeast extract, ammonium sulphate, and ammonium acetate were (Mahapatra et al. 2016) inoculated with standardised culture and incubated under optimal conditions as described above.

### 2.9 Effect of pH on β-glucosidase activity and stability of isolate PS1

Optimum pH was determined at 55°C for 5 min in various buffers: sodium acetate (0.05 M, pH 3–5), sodium phosphate (50 mM, pH 6–8), and Glycine-NaOH (50 mM, pH 9–10) containing 4 mM *4*-nitrophenyl-β-_D_-glucopyranoside. The pH stability was carried out by pre-incubating the enzyme in sodium phosphate buffer (50 mM, pH 6) for 4 h with aliquots sampled every 30 min. Residual activity was determined using the assay as per section 2.3. The enzyme in the optimum pH buffer with no incubation served as the control (100% activity).

### 2.10 Effect of temperature on isolate PS1 β-glucosidase activity and stability

The optimum temperature of the enzyme was determined in sodium phosphate buffer (0.05 M, pH 6.0) containing 4 mM *4*-nitrophenyl-β-_D_-glucopyranoside. The stability of the enzyme was determined by pre-incubating the enzyme at 55°C and 70°C in the absence of *4*-nitrophenyl-β-_D_-glucopyranoside for 4 h with aliquots sampled every 30 min. The residual activity was determined at 55°C for 5 minutes as per section 2.3. Residual activity was determined by using the enzyme in the optimum pH buffer with no incubation as the control (100% active).

### 2.11 SDS polyacrylamide gels

SDS-PAGE was carried out according to the procedure by Laemmli (1970). A 12% polyacrylamide was prepared. Electrophoresis was carried out at 50 V for 3 h and the gel was stained with Coomassie Brilliant Blue (More et al. 2011). The approximate molecular weight of the protein was determined from the bands that developed on the gel from the spectra multicolour broad range molecular weight markers (ThermoScientific, USA).

### 2.12 Native polyacrylamide gels

A native PAGE gel was used to detect the β-glucosidase enzyme. After electrophoresis, the gel was soaked in 0.05 M sodium acetate buffer (pH 5.0) for 10 minutes at room temperature, thereafter, incubated in 0.05 M sodium acetate buffer supplemented with 0.1% aesculin and 0.03% ferric chloride for 5 minutes at 55°C. After incubation, a black band should form corresponding to the protein band, thus confirming β-glucosidase activity (Kwon et al. 1994).

## 3. Results

### 3.1 Isolation, primary screening, and secondary screening of fungal isolates

A total of 46 isolates were obtained from three different sampling sites. Primary screening for β-glucosidase activity using aesculin revealed that 12 isolates had no precipitate indicating an absence of β-glucosidase activity, 34 displayed precipitation indicative of positive β-glucosidase activity as follows: 7 isolates displayed a slight precipitate indicating the presence of activity, 13 displayed a medium precipitate indicative of good activity, and 14 isolates displayed large precipitates indicating excellent β-glucosidase activity (Supplementary Figure and Table 1). All 14 excellent producers (C1; CB9; CB11, MS2; MS3; MS5; MS7; MB2; MB5; PS1; PB2; PB4; PB6; PB8) displayed large precipitate zones with the largest precipitate diameter of 75 mm for 10 isolates (C1; CB11; MS2; MS3; MS5; MB2; PB2; PB4; PB6; PB8) which displayed complete hydrolysis indicated by complete blackening of the entire surface of the substrate agar plates. The black precipitate and the zones of hydrolysis around the fungal growth are indicative of extracellular β-glucosidase and cellulase production, respectively (Bonciani et al. 2018).

**Table 1.** Identity of the seven fungal isolates after NCBI BLAST analysis.

	Description	Max score	Total score	Query cover	E-value	Identity	Accession number
C1	*Aspergillus japonicas* VIT-SB1	1018	2035	93%	0.0	100%	KC128815.1
C2	*Neofusicoccum parvum*_a_	1035	2055	99%	0.0	99.82%	MN180877.1
C4	*Meyerozyma guilliermondi* XQ9	841	1534	96%	0.0	90.22%	KY104257.1
MS8	*Trichoderma atroviride*	1081	2124	98%	0.0	99.49%	MG972798.1
PB8	*Lasiodiplodia iranensis*	965	1920	99%	0.0	100%	KY052971.1
PS1	*Chaetomella* sp. BBA70074	835	1651	98%	0.0	99.56%	AJ30196.1
MB5	*Neofusicoccum parvum*_b_	1035	2055	99%	0.0	99.82%	MN180877.1

**Figure 1.**
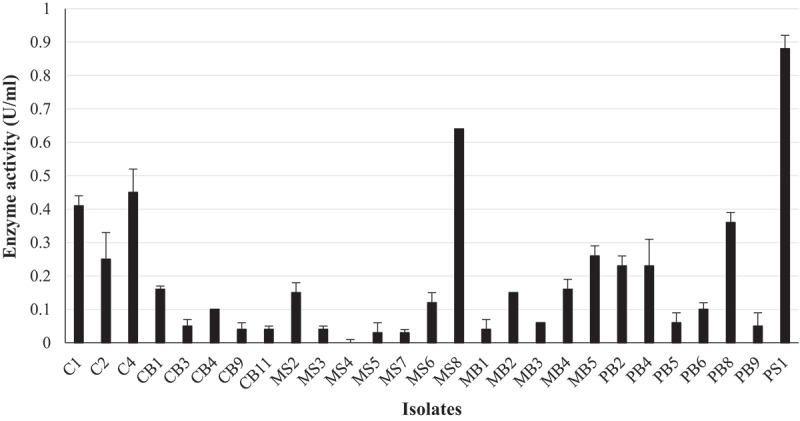
Quantification of β-glucosidase activity for the 27 fungal isolates with the largest precipitate zones from qualitative screening in the crude enzyme extracts produced at 30°C, 125 rpm and *4*-nitrophenyl-β-_D_-glucopyranoside as a substrate at OD_410 nm_,(Mean ± SD, N = 4).

**Figure 2.**
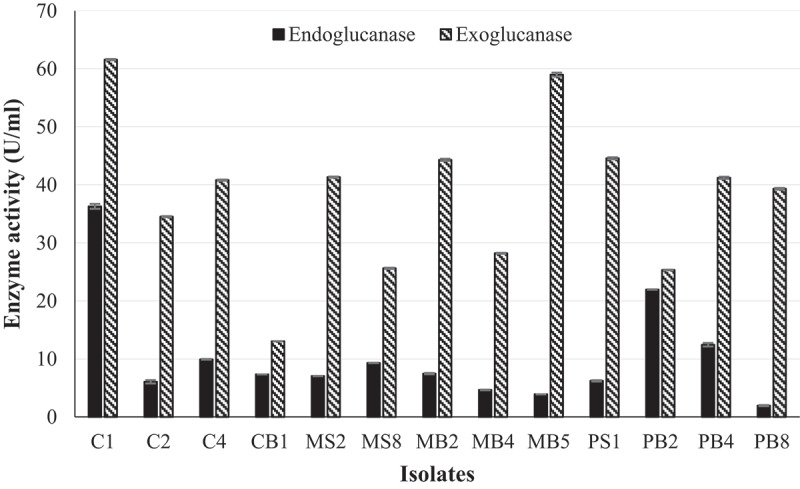
Quantification of endoglucanase and exoglucanase activity of the 13 isolates that displayed the highest β-glucosidase activity using the crude enzyme extract produced at 30°C, 125 rpm and the DNS assay with avicel and CMC as substrates, respectively at OD _540 nm_ (Mean ± SD, N = 4).

Fungal isolates MB2, MB5, PB4, and PB8 displayed a black pigment when grown on PDA. This made it difficult to distinguish between the black fungal pigment and the precipitate due to hydrolysis of the aesculin substrate. Therefore, secondary screening was performed to quantify β-glucosidase activity. The β-glucosidase activity of all 27 isolates displaying medium-to-large zones of precipitation indicative of enzyme activity from primary screening were quantified using *4*-nitrophenyl-β-_D_-glucopyranoside as a substrate (Figure 1). Of these, 13 isolates displayed (C1, C2, C4, CB1, MS2, MS8, MB2, MB4, MB5, PS1, PB2, PB4, PB8) enzyme activities between 0.4 U/ml and 0.9 U/ml, with isolate PS1 displaying the highest activity. All 13 isolates were then screened for endoglucanase and exoglucanase activity. Isolates C2, C4, MS8, PB2, and PB8 completely hydrolysed the CMC substrate, indicating good endoglucanase production (Supplementary Figure 1 and Table 2). Isolates C1, C2, MS8, MB2, MB4, PS1, PB2, and PB8 completely hydrolysed the avicel substrate indicated by complete clearing of the plate after qualitative screening methods compared to the control indicating excellent exoglucanase production (Supplementary Figure 1 and Table 2). Cellulase activity was then quantified for all 13 isolates, and the results in Figure 2 revealed that isolate C1 was the highest producer of both endoglucanases (36.3 U/ml) and exoglucanases (61.58 U/ml). The ability to produce β-glucosidases in the presence of glucose and the ability of the β-glucosidase enzyme to tolerate glucose were tested for the seven highest β-glucosidase producers (PS1; MS8; C4; C1; C2, MB5; PB8).

**Table 2.** Ratio of β-glucosidases to glucanases in the crude extracts of the fungal isolates.

Isolate	β-Glucosidase (U/ml)	Endoglucanase (U/ml)	Exoglucanase (U/ml)	β-Glucosidase: Endoglucanase: Exoglucanase
C1	0.4	36	61	1: 90: 153
C2	0.25	5	35	1: 20: 140
C4	0.45	10	40	1: 22: 89
CB1	0.25	5	12	1: 20: 48
MS2	0.25	5	40	1: 20: 160
MS8	0.65	9	28	1:13: 43
MB2	0.15	5	42	1: 33: 280
MB4	0.15	4	28	1: 27: 187
MB5	0.25	3	38	1: 12: 152
PS1	0.9	4	42	1: 4: 46
PB2	0.23	21	25	1: 91: 109
PB4	0.23	12	40	1: 52: 174
PB8	0.35	2	39	1: 6: 111

### 3.2 Identification of fungal isolates and phylogenetic analysis

The above seven fungal isolates were identified by sequencing of the ITS2 region of the 600 bp 18S ITS2 ribosomal RNA amplicons. Genomic DNA was successfully isolated from all seven isolates, and the 18S ribosomal RNA was amplified and the expected 600 bp amplicon was obtained. The BLAST analysis of the consensus sequences revealed the identities of all seven isolates. Isolate (C1) *Aspergillus japonicas*VIT-SB1, (C2) *Neofusicoccum parvum_a_*, (C4) *Meyerozyma guilliermondi* XQ9, (MS8) *Trichoderma atroviride*, (PB8) *Lasiodiplodia iranensis*, (PS1) *Chaetomella* sp. BBA70074, and (MB5) *Neofusicoccum parvum_b_*. Isolates C2 and MB5 displayed the same identities with different accession numbers indicating that they are different strains (Table 1). The phylogenetic tree displayed four clades, with nodes representing common ancestry (Figure 5). Several species were grouped in clade I, including *M. guilliermondi* (XQ9), *T. atroviride, C. raphigera, A. japonicas,* and *N. parvum*. Clade II only displays different *Chaetomella* sp. The *N. parvum_a_* and *N. parvum_b_* isolates surprisingly did not group together in one clade but were in clades III and IV, forming sister taxa with *N. parvum* strain 4 KF294004.1 and *N. parvum* MTZ41 KY052955.1 respectively. *Lasiodiplodia iranensis* RB31 and *N. parvum_a_* F7 have descended from the same common ancestor into two separate lineages. All the *A. japonicas* isolates are grouped together in clade I except for *A. japonicas* VIT-SB1 which formed sister clades with *Chaetomella* sp. ST001 and *M. guilliermondi* AP. MSU5.

### 3.3 Production of β-glucosidases in the presence of glucose

**Figure 3.**
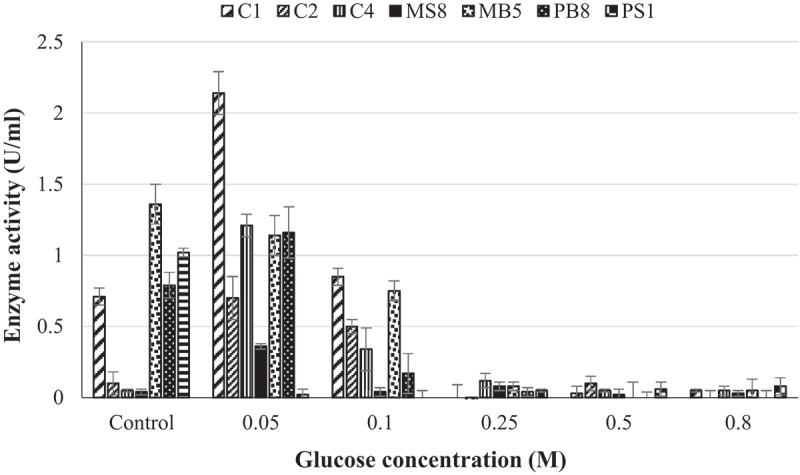
Effect of glucose on the production of β-glucosidases by (C1) *Aspergillus japonicas*, (C2) *Neofusicoccum parvum*_a,_ (C4) *Meyerozyma guilliermondi*, (MS8) *Trichoderma atroviride*, (MB5) *Neofusicoccum parvum*_b_, (PB8) *Lasiodiplodia iranensis*, and (PS1) *Chaetomella* sp. at 30°C, 125 rpm using *4*-nitrophenyl-β-_D_-glucopyranoside as a substrate at OD _410 nm_ (Mean ± SD, N = 4).

The effect of glucose on β-glucosidase production was determined to ensure that the enzyme produced is able to remain active in the presence of glucose. Production in the presence of 0.05 M glucose was enhanced for five isolates (C1, C2, C4, MS8, and PB8) by greater than 50% and decreased marginally at a higher glucose concentration (0.1 M). From glucose concentrations ≥0.25 M the enzyme production decreased by greater than 70% for all seven isolates (Figure 3).

### 3.4 Glucose tolerance assays

**Figure 4.**
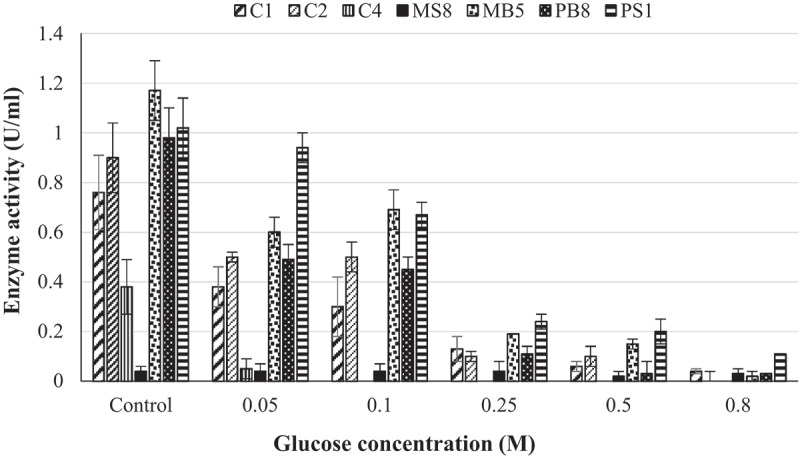
Glucose tolerance of crude β-glucosidases produced by (C1) *Aspergillus japonicas*, (C2) *Neofusicoccum parvum*_a,_ (C4) *Meyerozyma guilliermondi*, (MS8) *Trichoderma atroviride*, (MB5) *Neofusicoccum parvum*_b_, (PB8) *Lasiodiplodia iranensis*, (PS1) *Chaetomella* sp., at 30°C, 125 rpm using *4*-nitrophenyl-β-_D_-glucopyranoside as a substrate at OD _410 nm_ (Mean ± SD, N = 4).

The glucose tolerance studies indicated that five of the seven isolates, namely *Aspergillus japonicas* (*A. japonicas*), *N. parvum*_a_, *N. parvum*_b_, *Lasiodiplodia iranensis* (*L. iranensis*), and *Chaetomella* sp. retained greater than 50% of their β-glucosidase activities in the presence of 0.05 M glucose. *Meyerozyma guilliermondi* (*M. guilliermondi*) and *Trichoderma atroviride* (*T. atroviride*) lost almost all their activity (Figure 4). The three isolates that retained the highest β-glucosidase activity in the presence of 0.05 M glucose were *Chaetomella* sp. (92%), *N. parvum*_a_ (55%), and *N. parvum*_b_(51%). *Chaetomella* sp. displayed the highest tolerance to glucose and retained 11% of activity, *N. parvum*_a_ displayed no activity, and *N. parvum*_b_ 1% β-glucosidase activity in the presence of 0.8 M glucose. These isolates were taken forward for optimisation of enzyme production by one variable at a time experiments. The optimised crude enzyme extract of *Chaetomella* sp. retained 31% β-glucosidase activity in the presence of 0.8 M glucose, this is 20% more enzyme activity retained compared to the initial crude extract whilst the optimised crude extract of isolates *N. parvum*_a_ and *N. parvum*_b_ did not show an increase in tolerance to glucose when compared to the initial crude extract.

**Figure 5.**
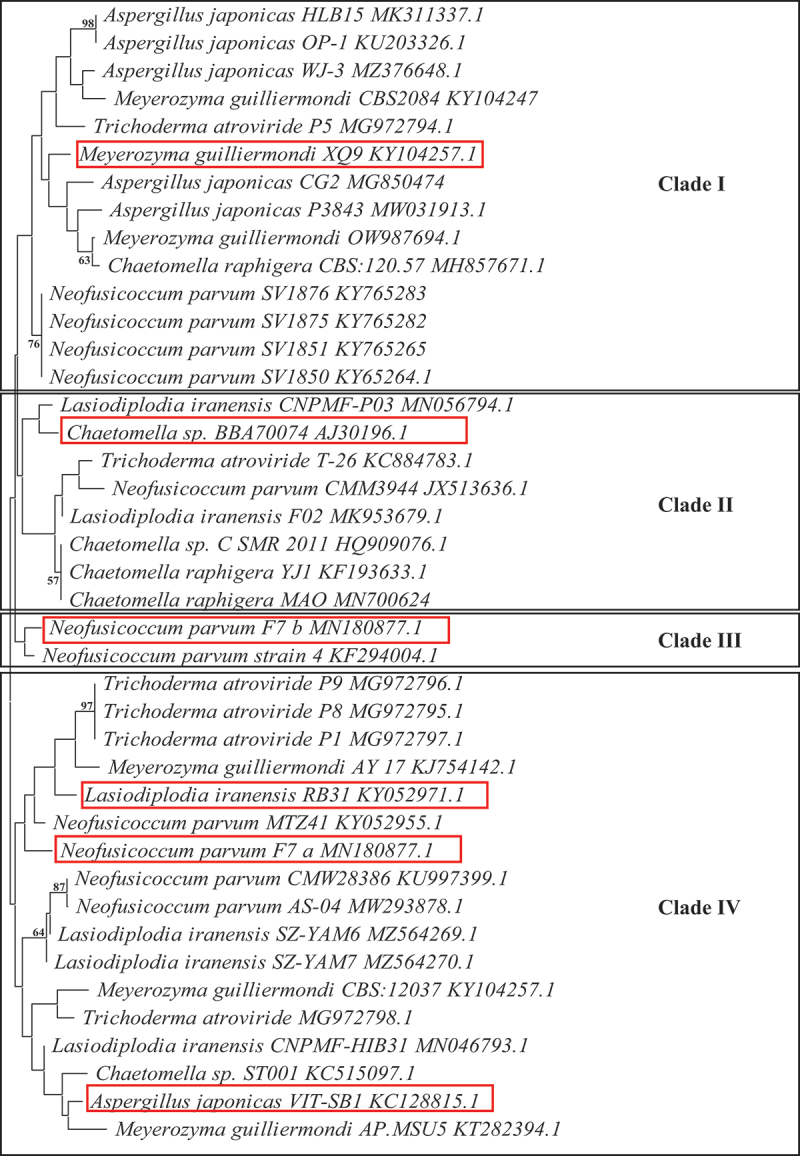
Phylogenetic analysis of the seven fungal isolates identified based on the alignment of their ITS2 18S rRNA nucleotide sequences with Mega 11. The microbial species, strain name and accession number are presented. The numbers on the branches indicate their bootstrap support.

### 3.5 *Optimisation of enzyme production by* N. parvum*_a_*, N. parvum*_b_ and* Chaetomella *sp*

**Figure 6.**
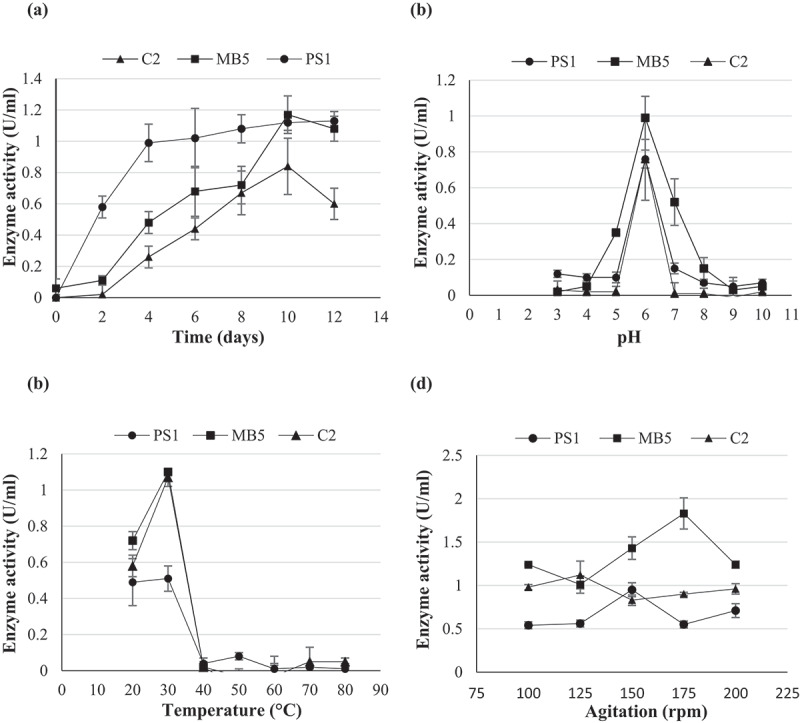
Optimal time of incubation (a) optimum pH (b) temperature (c) and agitation (d) using one factor at a time experiments for the production of β-glucosidases by isolates (C2) *Neofusicoccum parvum*_a_, (MB5) *Neofusicoccum parvum*_b_, and (PS1) *Chaetomella* sp. using the crude enzyme extracts and *4*-nitrophenyl-β-_D_-glucopyranoside as substrate at OD _410 nm_ (Mean ±SD, N = 4).

**Figure 7.**
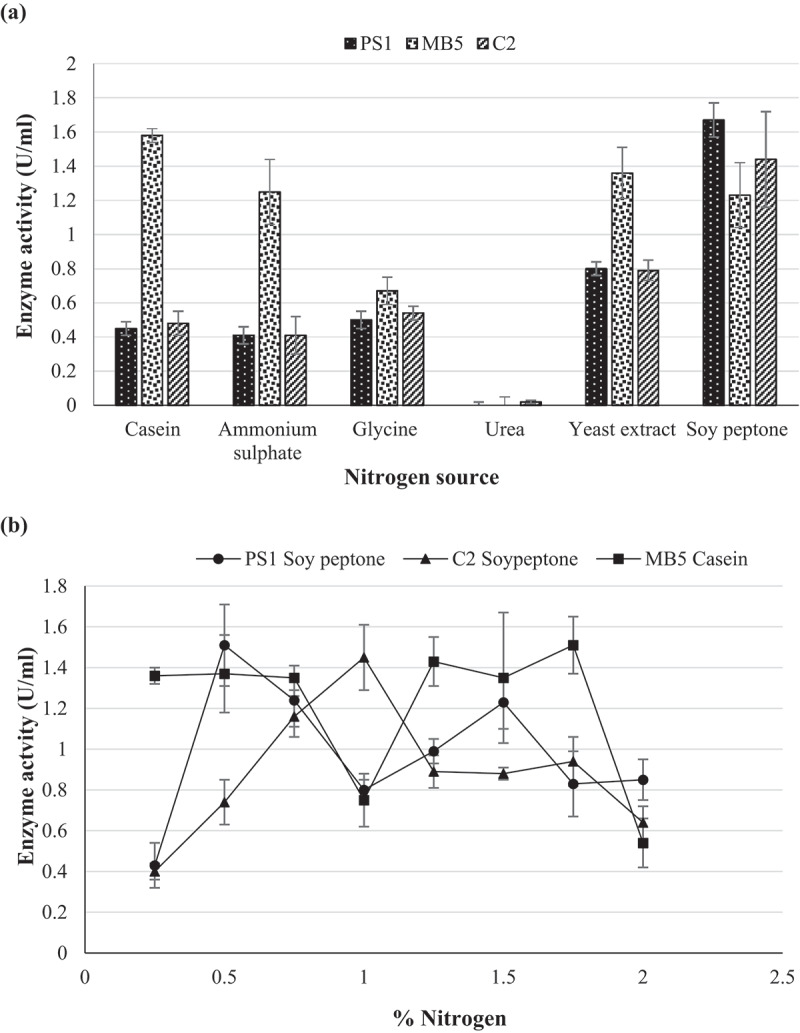
Optimum (a) nitrogen source and (b) nitrogen concentration for the optimal production of β-glucosidase by isolates (C2) *Neofusicoccum parvum*_a_, (MB5) *Neofusicoccum parvum*_b_, and (PS1) *Chaetomella* sp. using the crude enzyme extracts and *4*-nitrophenyl-β-_D_-glucopyranoside as substrate at OD_410 nm_ (Mean ±SD, N = 4).

**Figure 8.**
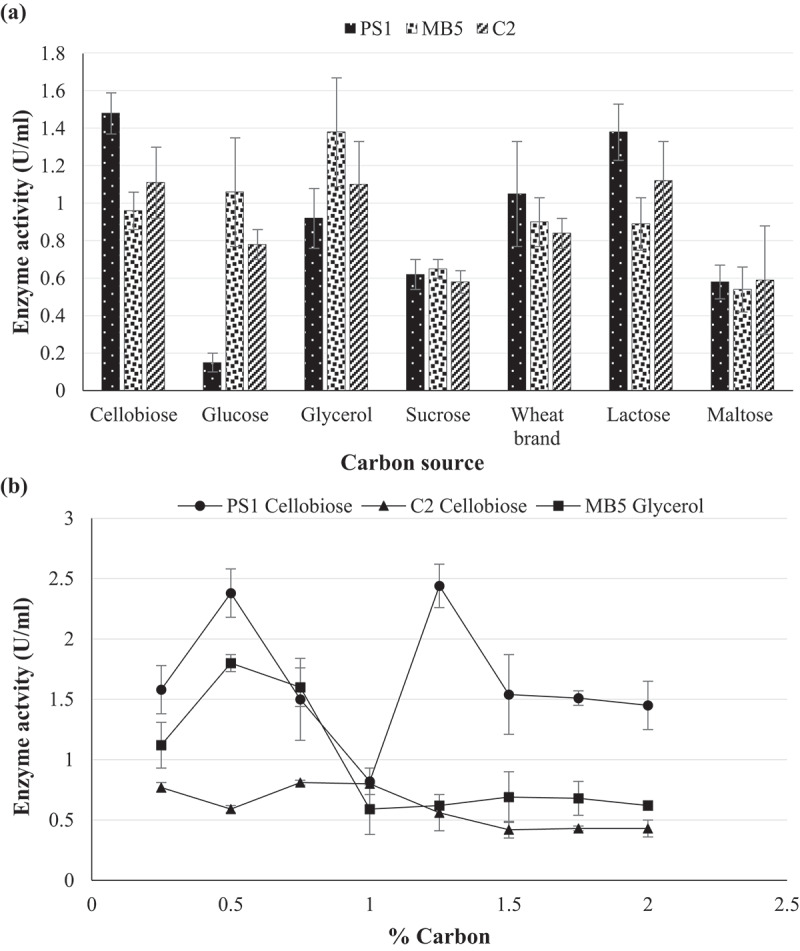
Optimum (a) Carbon source and (b) Carbon concentration for the optimal production of β-glucosidase by isolates (C2) *Neofusicoccum parvum*_a_, (MB5) *Neofusicoccum parvum*_b_, and (PS1) *Chaetomella* sp. using the crude enzyme extracts and *4*-nitrophenyl-β-_D_-glucopyranoside as substrate at OD_410 nm_ (Mean ±SD, N = 4).

Optimisation of enzyme production was achieved by determining optimal growth parameters so as to achieve maximal enzyme levels from fermentation, thus decreasing costs. The optimal incubation time for the production of β-glucosidase by *Chaetomella* sp. was 6 days, *N. parvum*_a_ 9 days, and *N. parvum*_b_ 10 days (Figure 6a). Maximum production was obtained at pH 6.0 and 30°C for all three isolates (Figures 6b and c). The agitation speeds of 150, 125, and 175 rpm yielded optimal enzyme titres for *Chaetomella* sp., *N. parvum*_a,_ and *N. parvum*_b_, respectively (Figure 6d). Casein and glycerol were the best nitrogen and carbon sources for *N. parvum*_b_ yielding an enzyme activity of 1.8 U/ml in 1.75% and 0.5% nitrogen and carbon, respectively (Figures 7 and 8). Isolates *N. parvum*_a_ and *Chaetomella* sp. displayed optimal β-glucosidase production with supplementation of soy peptone and cellobiose, respectively, displaying enzyme activities of 0.80 U/ml in 1% soy peptone and 1% cellobiose and 2.44 U/ml in 0.5% soy peptone and 1.25% cellobiose, respectively (Figures 7 and 8). *Chaetomella* sp. produced the highest β-glucosidase activity of 2.44 U/ml (Figure 8).

### 3.6 Polyacrylamide gel electrophoresis

**Figure 9.**
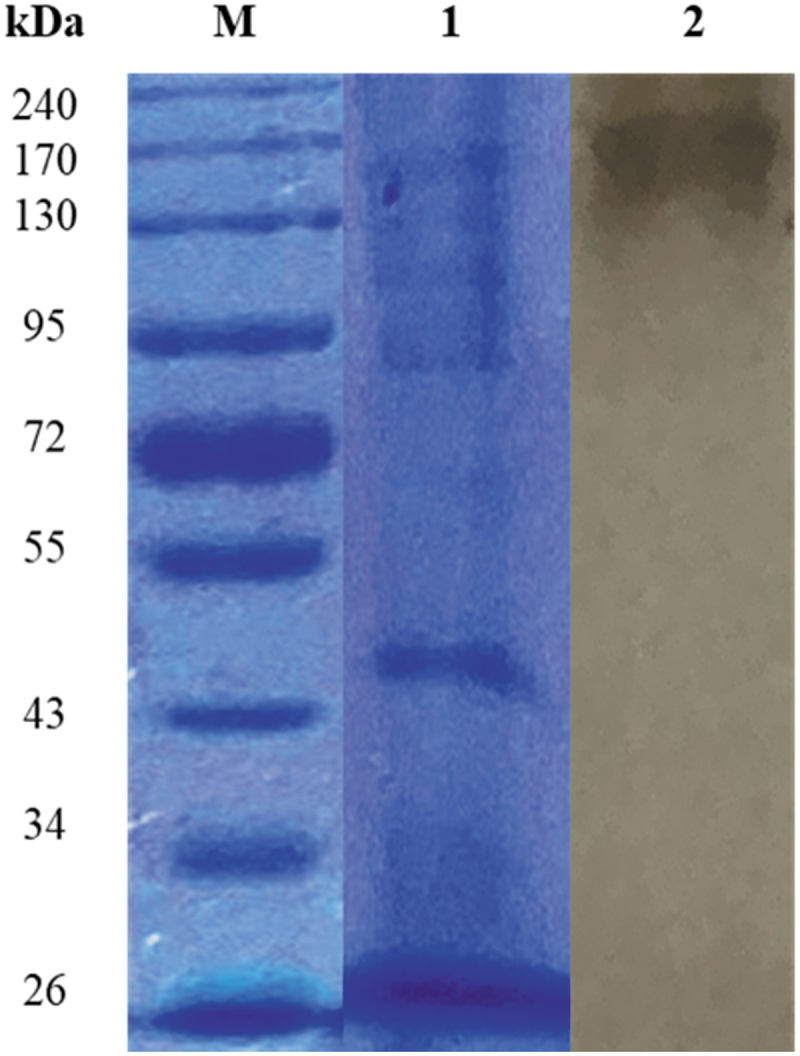
SDS and Native PAGE of (PS1) *Chaetomella* sp. crude β-glucosidase extract produced under optimal conditions (Day 12, 3°֯C, 150 rpm, 0.5% soy peptone, 1.25% cellobiose). Lanes M: spectra multicolour broad range molecular weight marker (ThermoScientific, USA), 1: crude enzyme, and 2: crude enzyme displaying β-glucosidase activity.

The SDS-PAGE gel of the crude β-glucosidase revealed multiple-protein bands in the crude extract. Zymography using non denaturing gel electrophoresis revealed a 170 kDa protein subunit displaying β-glucosidase activity (Figure 9).

#### 3.6.1. *Effect of pH and temperature on the activity of crude β-glucosidase enzymes produced by Chaetomella* sp. *BBA70074*

**Figure 10.**
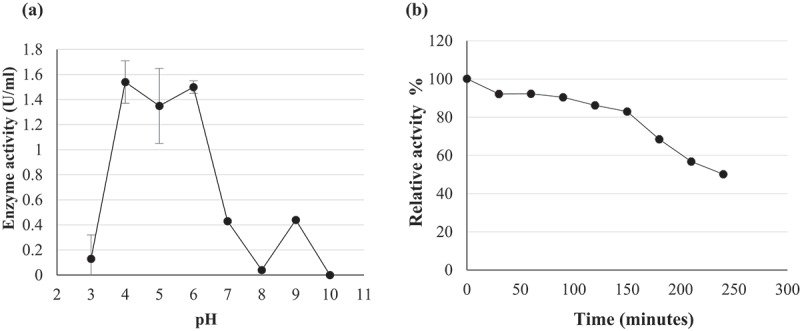
(a) pH optima and (b) stability at pH 6 of (PS1*) Chaetomella* sp. β-glucosidase crude extract produced under optimal conditions (Day 12; 30°C; 150 rpm; 0.5% soy peptone; 1.25% cellobiose) and assayed using *4*-nitrophenyl-β-_D_-glucopyranoside as substrate at 55°C and OD_410 nm_ (Mean ±SD, N = 2).

The pH optima for β-glucosidase activity of *Chaetomella* sp. BBA70074 was pH 4.0 and 6.0 (Figure 10a). β-Glucosidase displayed maximal activity at 70°C in pH 6.0 buffer (Figure 10a) indicating a thermophilic enzyme. pH and temperature stability studies were conducted focusing on the parameters and levels that were similar to those required for industrial enzymes.

#### 3.6.2. *pH and temperature stability of crude Chaetomella* sp. *β-glucosidase enzymes*

**Figure 11.**
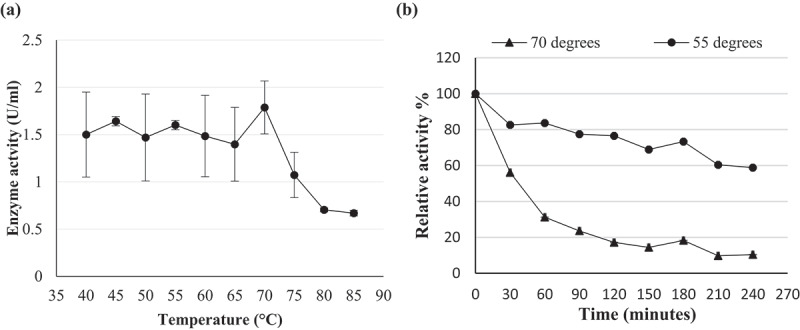
(a) Temperature optima and (b) stability at 55°C and 70°C of (PS1) *Chaetomella* sp. crude β-glucosidase extract produced under optimal conditions (Day 12, 3°֯C, 150 rpm, 0.5% soy peptone, 1.25% cellobiose) and assayed using *4*-nitrophenyl-β-_D_-glucopyranoside as substrate at 55°C and OD_410 nm_ (Mean ±SD, N = 2).

The enzyme was fairly stable for 150 minutes, retaining 80% activity at pH 6.0. At 180 minutes the β-glucosidase activity started to decline with the enzyme retaining only 50% activity at 240 minutes (Figure 10b). At 70°C, the enzyme was unstable and lost approximately 40% activity in the first 30 minutes with a further 30% activity lost after 60 minutes and only 10% activity remaining at 240 minutes (Figure 11b). Since the enzyme was not stable at its optimal temperature, thermal stability was tested at the next optimal temperature point (55°C). Better stability was observed at 55°C compared to 70°C. The enzyme was fairly stable retaining 80% activity within the first 90 minutes, from 120 to 180 minutes a decline in enzyme activity and at 240 minutes the enzyme retained 60% activity (Figure 11b).

## 4. Discussion

In this study, a total of 46 isolates were obtained from soil, tree, and bark samples from three different sites and screened for β-glucosidase activity and 13 of these were screened for glucanase activity by primary screening methods. The colour intensity of precipitation and the zones of hydrolysis in primary screening display no correlation with the enzyme activities obtained in secondary screening. It is, therefore, important to utilise both primary and secondary screening methods as qualitative screening methods are less sensitive compared to quantitative methods (Bonciani et al. 2018).

*A. japonicas* was the highest cellulase producer with β-glucosidases with promise as a native producer of cellulase cocktails for the degradation of cellulosic biomass in industrial applications. It produced all three cellulase enzymes with glucose tolerant β-glucosidases (Table 2). Rani et al. (2014) reported that the commercial *T. reesei* cellulase cocktail with ratios of β-glucosidase: endoglucanase: exoglucanase of <1%: 18%: 72% resulted in hydrolysis of cellulosic material; however, this cocktail had to be supplemented with β-glucosidases from *A.niger* as the β-glucosidase in the *T. reesei* cocktail was not at an appropriate concentration and was inhibited by high levels of glucose.

Based on the different effects of glucose on β-glucosidase activity in this study those from isolates *M. guilliermondi* and *T. atroviride* can be grouped into group one whilst all of the others belong to group two (Cao et al. 2015). *Chaetomella* sp. displayed the highest tolerance to glucose retaining 92% and 11% of its activity at low 0.05 M and high 0.8 M glucose concentrations, respectively. Cao et al. (2015) reported 10% retention of activity in 0.6 M glucose, which indicates that isolate *Chaetomella* sp. has great promise as its β-glucosidase retained 11% activity in 0.8 M glucose, which is a higher concentration of glucose.

Studies have reported the isolation of β-glucosidases and cellulases from *Chaetomella* sp. and *M. guilliermondi*; however, very low activities were observed (Sari et al. 2017; Kao et al. 2019; Zhang et al. 2021). Species of *Aspergillus* and *Trichoderma* display great potential for biotechnological processes. A study by Decker et al. (2000) reported β-glucosidases from *A. japonicus* with enzyme activity of 29.5 U/ml; however, the tolerance to glucose was not tested. In another study, Valappil et al. (2019) reported glucose tolerant β-glucosidases from *A. unguis* that retained less than 10% β-glucosidase activity in 0.5 M of glucose. Cellulases including β-glucosidases from *A. niger* and species of *Trichoderma* are generally regarded as yardsticks for commercial production; however, the proportion of β-glucosidases in these cellulase cocktails is meagre, and they require supplementation with β-glucosidases. Industrial-level scale-up of the process is inefficient as naturally-occurring cocktails do not contain optimal ratios and specific activities of all three components. *T. reesei* has already been commercialised and utlised in biotechnological applications (Ahmed et al. 2017). Two other *Trichoderma* strains, namely, *T. atroviridae* and *T. virens* display potential for commercialisation.

The whole-genome sequence of *T. atroviride* and *T. virens* is incomplete, but that of *T. reesei* has been completed. The sequence data shows that the genome consists of few genes encoding hemicellulolytic and cellulolytic enzymes (Schmoll et al. 2016). The whole-genome sequence will enable scientists to obtain the β-glucosidase gene sequences, which may be utilised to produce recombinant enzymes with potentially enhanced β-glucosidase activity. Makraki et al. (2020) produced a recombinant plasmid by cloning the *T. reesei* (*Trbgl2*, AB003110) gene into a pET vector. The appropriate codon in the recombinant plasmid was then substituted with the E367Q gene fragment by site directed mutagenesis. The resulting mutant displayed a 188% increase in activity. Cao et al. (2015) isolated a *Bgl*6 clone from a 260 Mb metagenomic library with an open reading frame of 1371 bp encoding 456 amino acids displaying homology similar to glucose tolerant β-glucosidases from the GH1 family.

The isolates under study were identified by NCBI nucleotide blast and the identities of the respective unknown isolates are represented in Table 1. All seven isolates belong to the phylum Ascomycota, which represents the most diverse group of fungi. The principal characteristic of this group includes an ascus, which is a sac-like structure that contains ascopores (Naranjo-Ortiz and Gabaldón 2019). Phylogenetic analysis plays an important role in understanding the evolution of species, populations, and genes (Figure 5). Evolutionary relationships between species are represented in phylogenetic trees (Chen and Zhuang 2017). *Neofusicoccum parvum*_a_ and *N. parvum*_b_ isolated from two different areas descended from the same common ancestor into clades IV and III, respectively. These two isolates were morphologically identical except for a different pigmentation pattern. The fungal species *A. japonicas* and *N. parvum_a_* isolated from the same area belong to the same clade indicating that they share a common ancestor and similar characteristics but are from different genera. These two scenarios are an indication that the nucleotide sequences encoding genes in different species may be influenced by environmental factors (Taylor et al. 2015). *N. parvum*_a_, *N. parvum*_b,_ and *L. iranensis* are known endophytic fungi; however, this is the first report of the production of β-glucosidases and cellulases from these isolates. *N. parvum* is also a plant pathogen on different trees in various areas worldwide (Chan et al. 2021) while *L. iranensis* inhabits eucalyptus, citrus, and many other plants as an endophyte (Du et al. 2020). In this study, two isolates were identified as *N. parvum*, however, they were isolated from two different areas and although both isolates displayed similar morphologies, their black pigmentation on PDA differed (Supplementary Figure 1). *N. parvum*_a_ displayed a central black circular pigmentation pattern on the bottom of the plate whilst *N. parvum*_b_ displayed a patchy black pigmentation on both sides of the plates. In addition, the biochemical character of the isolates differed in one variable at a time experiments except for temperature and pH. They displayed different preferences for nutrients (carbon and nitrogen). *N. parvum*_b_ displayed higher tolerance to glucose compared to *N. parvum*_a_ with 0.2% and 0.1% activity retained in 0.5 M glucose, respectively. This change may be the result of different selective pressures caused by environmental differences to which they adapted, thus culminating in different growth parameters for optimal β-glucosidase production. This phenomenon is not surprising and can be attributed to Darwin’s law of natural selection, which states that “A population in equilibrium with its environment under natural selection will have a phenotype which maximises the fitness locally” (Haufe 2012).

*Chaetomella* sp. displayed the highest enzyme production on day 12; however, day 6 was selected as the optimal day of production as a t-test displayed a *p*-value >0.5 indicating that difference in enzyme levels between days 6 and 12 was not significant; however, the reduced production time will translate to production cost savings. *N. parvum*_a_ and *N. parvum*_b_ also required long incubation periods for optimal enzyme production and an optimal pH and temperature of six and 30°C, respectively. Optimal β-glucosidase activity was also reported to occur after incubation periods longer than 5 days by Gao et al. (2012, 2016, Ahmed et al. (2017) and Kao et al. (2019). The optimal pH of 6 and temperature of 30°C are expected as a study by Agarwal *et al*. (2014) reported optimal β-glucosidase production at the same pH and temperature. *Chaetomella* sp. and *N. parvum*_a_ displayed optimal production of β-glucosidases when supplemented with soy peptone and cellobiose as carbon and nitrogen sources, respectively. This is not remarkable as many studies report variations in carbon and nitrogen source preferences by different fungi (Gao et al. 2012; Sørensen et al. 2014; Ahmed et al. 2017). A study by Mahapatra et al. (2016) reported the optimal production of β-glucosidase enzymes from *A. niger* using glycerol as a carbon source. Soy peptone has also been reported for optimal β-glucosidase production by *Chaetomium thermophilum* (Mahapatra et al. 2016). Other carbon sources preferred by microorganisms that were not tested in this study include avicel, pectin, and quercetin and nitrogen sources include beef extract, L- asparagine, and tryptone (Ahmed et al. 2017).

Hydrolysis of the lignocellulosic biomass requires a basic pH of 5–9 and thermophilic temperatures between 50°C and 85°C (Kao et al. 2019). *Chaetomella* sp. displayed an optimal pH and temperature at pH 6 and 70°C, respectively. Two other studies reported pH and temperature optima within the ranges (pH 5.0–9.0) and (50–70°C) indicating their thermophilic nature (Karnchanatat et al. 2007; Karami et al. 2020). Karami et al. (2020) reported a fairly good stability of β-glucosidase after 1 hour at 70°C, whereas the enzyme in the current study was not stable at 70°C; however, thermal stability was tested at 55°C and revealed that the enzyme was stable for 120 minutes indicating that the β-glucosidase enzyme is still suitable for application in cellulose hydrolysis processes.

The present study focused on searching for a novel cellulase producer with glucose tolerant β-glucosidase with optimal specific activities, which would be more cost-effective in scaling up. The strains isolated displayed promising activity for both cellulases and glucose tolerant β-glucosidases. *A. japonicas* displayed promising activity as a prospective producer of a cocktail of all three enzymes for application in industry for the hydrolysis of cellulose. In addition to high glucanase activity, it also has a promising ratio of β-glucosidase:exoglucanase:endoglucanase. The β-glucosidase from this isolate also showed tolerance to glucose; however, the *Chaetomella* sp. and *N. parvum_b_* isolates displayed higher tolerance to glucose compared to that of *A. japonicas*. These isolates may also be suitable for application in industry as producers of glucose tolerant β-glucosidases for supplementation of existing cellulase cocktails for the hydrolysis of cellulose (Karami et al. 2020).

Future studies include optimisation of enzyme production using one variable at a time experiments, Plackett Burman, Response Surface Methodology, purification, and characterisation of the enzymes of interest.

## Supplementary Material

Supplemental Material
