# Supplementary material for: Screening for cellulases and preliminary optimisation of glucose tolerant β-glucosidase production and characterisation
Source: Mycology. 2022 Dec 17;14(2):91–107. doi: 10.1080/21501203.2022.2155261 (PMC10161942; doi:10.1080/21501203.2022.2155261)
Supplement: Supplemental Material [file TMYC_A_2155261_SM0940.docx]

Table 1: Qualitative screening for β-glucosidase activity using substrate agar plates 1% Esculin and 0.05% ferric citrate

| **Isolate** | **Score** |
| --- | --- |
| **C1** | +++ |
| **C2** | ++ |
| **C3** | - |
| **C4** | ++ |
| **C5** | - |
| **C6** | - |
| **C7** | + |
| **CB1** | ++ |
| **CB2** | + |
| **CB3** | ++ |
| **CB4** | ++ |
| **CB5** | + |
| **CB6** | + |
| **CB7** | - |
| **CB8** | + |
| **CB9** | +++ |
| **CB10** | - |
| **CB11** | +++ |
| **MS1** | + |
| **MS2** | +++ |
| **MS3** | +++ |
| **MS4** | ++ |
| **MS5** | +++ |
| **MS6** | ++ |
| **MS7** | +++ |
| **MS8** | ++ |
| **MB1** | ++ |
| **MB2** | +++ |
| **MB3** | ++ |
| **MB4** | ++ |
| **MB5** | +++ |
| **PS1** | +++ |
| **PS2** | - |
| **PS3** | - |
| **PS4** | + |
| **PB1** | - |
| **PB2** | +++ |
| **PB3** | - |
| **PB4** | +++ |
| **PB5** | ++ |
| **PB6** | +++ |
| **PB7** | - |
| **PB8** | +++ |
| **PB9** | ++ |
| **PB10** | - |
| **PB11** | - |

Key: No zone (-), 1-30 mm (+), 31-50 mm (++), > 50 mm (+++)

Table 2: Qualitative screening for endoglucanase and exoglucanase activities using substrate agar plates with 1% CMC and avicel, respectively.

| **Isolate** | **Endoglucanase Score** | **Exoglucanase Score** |
| --- | --- | --- |
| C1 | - | ++ |
| C2 | ++ | ++ |
| C4 | ++ | + |
| CB1 | - | - |
| MS2 | + | + |
| MS8 | ++ | ++ |
| MB2 | + | ++ |
| MB4 | - | ++ |
| MB5 | + | - |
| PS1 | ++ | ++ |
| PB2 | ++ | ++ |
| PB4 | - | - |
| PB8 | ++ | ++ |

Key: No zone (-), 1-2 cm (+), 1-2 cm, > 2 cm (++)
